# Identifying protein complexes from interaction networks based on clique percolation and distance restriction

**DOI:** 10.1186/1471-2164-11-S2-S10

**Published:** 2010-11-02

**Authors:** Jianxin Wang, Binbin Liu, Min Li, Yi Pan

**Affiliations:** 1 School of Information Science and Engineering, Central South University, Changsha 410083, China; 2 Department of Computer Science, Georgia State University, Atlanta, GA30303-4110, USA

## Abstract

**Background:**

Identification of protein complexes in large interaction networks is crucial to understand principles of cellular organization and predict protein functions, which is one of the most important issues in the post-genomic era. Each protein might be subordinate multiple protein complexes in the real protein-protein interaction networks. Identifying overlapping protein complexes from protein-protein interaction networks is a considerable research topic.

**Result:**

As an effective algorithm in identifying overlapping module structures, clique percolation method (CPM) has a wide range of application in social networks and biological networks. However, the recognition accuracy of algorithm CPM is lowly. Furthermore, algorithm CPM is unfit to identifying protein complexes with meso-scale when it applied in protein-protein interaction networks. In this paper, we propose a new topological model by extending the definition of *k*-clique community of algorithm CPM and introduced distance restriction, and develop a novel algorithm called CP-DR based on the new topological model for identifying protein complexes. In this new algorithm, the protein complex size is restricted by distance constraint to conquer the shortcomings of algorithm CPM. The algorithm CP-DR is applied to the protein interaction network of *Sacchromyces cerevisiae* and identifies many well known complexes.

**Conclusion:**

The proposed algorithm CP-DR based on clique percolation and distance restriction makes it possible to identify dense subgraphs in protein interaction networks, a large number of which correspond to known protein complexes. Compared to algorithm CPM, algorithm CP-DR has more outstanding performance.

## Background

With the Human Genome Project implement successfully, the biomedical research enters the post-genome era. In the new era, one of the most important challenges is to systematically analyze and comprehensively understand how the proteins accomplish the life activities by interacting with each other [1]. It plays an important role in predicting the protein functions and understanding specific biological processes that identify protein complexes from large-scale protein-protein interaction networks. In recent years, the development of large-scale interaction prediction techniques results a large number of protein-protein interaction (PPI) data. Moreover, a large number of algorithms for detecting protein complexes from protein-protein interaction networks have emerged. According to whether the algorithm could identify overlapping protein complexes, these algorithms can be classed into two types, Non-overlapping Clusters Detecting Algorithms and Overlapping Clustering Identifying Algorithms.

The basic idea of Non-overlapping Clustering Algorithms is that each protein belongs to one and only one protein complex in large-scale protein-protein interaction network. King *et al.* proposed the Restricted Neighborhood Search Clustering (RNSC) algorithm which aimed at exploring the best partition of networks by using a cost function [2]. In addition, there are some typical Non-overlapping Clustering Algorithms. For example, Hartuv and Shamir used the minimum cut to remove edges recursively and developed a divisive algorithm HCS for mining highly connected clusters in networks [3], Girvan and Newman developed a divisive algorithm G-N based on the edge betweenness [4], Newman *et al.* proposed a fast agglomerative algorithm based on greedy strategy [5]. In recent years, some researchers extend the G-N algorithm, for instance, Radicchi *et al* gave a new self-contained algorithm [6] and Luo *et al* developed an agglomerative algorithm MoNet [7] and so on. In the real protein-protein interaction networks, however, protein complexes are usually overlapping, that is to say, some proteins may be subordinate more than one complex simultaneously [8]. Therefore, the researches on identification algorithm in mining overlapping protein complexes are more significance [9].

In recent years, a variety of algorithms extend the G-N algorithm could be employed to analyze the overlapping structures of the large-scale complex networks, including protein-protein interaction networks. The representative algorithms are Cluster-Overlap Newman Girvan Algorithm (CONGA) [10], the betweenness-based decomposition method (BCe) [11] and the Fuzzy Cluster algorithm [12]. Gregory *et al.*[10] discussed the edge betweenness centrality measure and developed a new algorithm CONGA, which decompose networks into arbitrary quantity overlapping structures, to discover overlapping communities in networks. By computing splitting betweenness, the algorithm CONGA determined when to split vertices, what vertex to split and how to split them. Because of the time complexity *O(m^3^)* relational closely to the scale of edges in network, the efficiency of algorithm CONGA is considerably low. A novel algorithm BCe based on edge betweenness and vertex betweenness obtain overlapping structures by choosing the similarity threshold values between vertices pairs. In the Fuzzy Clustering algorithm [12], Zhang *et al.* analyze the overlapping structures based on external input parameter which indicate an upper bound of the community quantity, while it is a considerably challenge that the input parameter absolutely equal to the number of complex in real protein-protein interaction networks. Furthermore, according to the *Centrality-Lethality Rule* generally existing in protein-protein interaction networks, Li *et al.*[14] developed a graph splitting and reduction model, and an original algorithm OMFinder for identifying overlapping functional modules based on the developed model is proposed. In algorithm OMFinder, the proteins are divided into two classes of high-degree and low-degree nodes, constrain only the high-degree nodes could subordinate to multiple protein complexes. Comparing to other approaches of detecting overlapping complexes, many significant overlapping protein complexes with various topologies could be discovered effectively by this algorithm with low discard rate. However, this algorithm only simply deal with the subgraphs, which decomposed from the protein-protein interaction network, containing high-degree proteins and the ones that include low-degree proteins

With specialized research in the overlapping structure of large-scale complex network, a powerful algorithm for finding protein complexes and exploring the general characteristics of complex networks in biology based on clique percolation has been recently developed by Palla *et al.,* named Clique Percolation Method (CPM) [15]. In addition, a software tool of detecting overlapping clusters CFinder is developed based on this algorithm CPM [8]. As an effective algorithm on identifying overlapping module structures, algorithm CPM has a wide range of application in social networks and biological networks [9,16], while its recognition accuracy is too low and unfit to identifying protein complexes with meso-scale when it applied in protein-protein interaction networks. Generally speaking, results of algorithm CPM are highly correlated to the value of the clique percolation parameter *k.* The smaller values of k correspond to the more excessive large subgraphs of high density. In order to conquest these shortcomings, an algorithm called CP-DR (Clique Percolation Method based on Distance Restriction) for identifying protein complexes based on clique percolation and distance restriction is proposed in this paper. In this algorithm, the scale of protein complex is restricted by distance constraint. The experiment results show that algorithm CP-DR can detect a large number of protein complexes with specific biological significance and biological functions more effectively, more precisely and more comprehensively.

## The Proposed Algorithm

### Algorithm CPM

**Figure 1:**
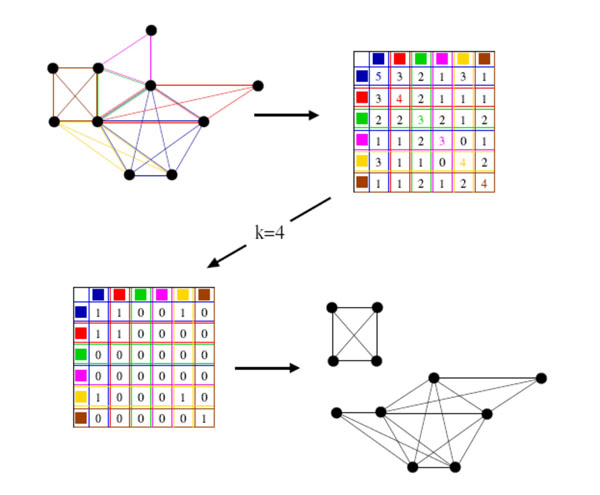
**A simple illustration of the extraction of the *k*-clique-communities at *k* = 4 using the clique-clique overlap matrix**[15]. Top left picture shows the graph in which the different cliques are marked by different colors. The according clique-clique overlap matrix is shown in the top right corner. To obtain the *k*-clique-communities at *k* = 4, algorithm CPM delete the off-diagonal elements that are smaller than 3 and also the diagonal elements that are smaller than 4, resulting in the matrix shown in the bottom left of the figure. The connected components (the *k*-clique-communities) corresponding to this matrix are shown in the bottom right.

Palla *et al.* proposed the algorithm CPM based on clique percolation method [15]. The underlying idea of this method is the concept of a *k*-clique community which was defined as the union of all *k*-cliques (complete subgraphs of size *k)* that can be reached from each other through a series of adjacent *k*-cliques (where adjacency means sharing *k*-1 vertices). The *k*-clique community can be considered as a usual module (community, cluster or complex) because of its dense internal links and sparse external linkage with other part of the whole network. All of *k*-cliques of network can be received by iterative recursion, then construct the overlap matrix of these *k*-cliques. Finally, a number of *k*-clique communities are discovered by analysis the overlap matrix. As a simple illustration, Fig.1 shows the steps of the extraction of the *k*-clique-communities at *k* = 4 using the clique-clique overlap matrix.

As is known to all, the result of algorithm CPM associated closely with the value of clique percolation parameter *k.* Generally speaking, the larger value of *k* chose, the smaller size of *k*-clique communities of higher density would be obtained. And it is no doubt that vertices are relatively dense linked internal each *k*-clique community. Although the algorithm CPM analysis the overlapping modular structure of society and biology is effective, the drawback is protein complexes quantity identified by this algorithm limited. Especially, protein complexes quantity is fewer when the relatively large *k* value chose. The large-scale *k*-clique communities usually correspond to small *k* values, that is to say, the smaller value of parameter *k* selected, the larger size of *k*-clique communities have. We applied the CPM method to yeast protein-protein interaction network and detected interesting protein complexes which might overlap each other. When using *k*=4, taking into account the basic topological unit as 4-clique, there is a large identified protein complex containing 348 vertices and 2499 pairs interactions. Meanwhile, there is an excessive huge protein complex detected with 865 vertices and 4508 pairs interactions when choosing *k*=3*.* As the previous examples, the scale of *k*-clique communities is far greater than scales of *k*-cliques and sparse *k*-clique chain.

### Algorithm CP-DR

**Figure 2:**
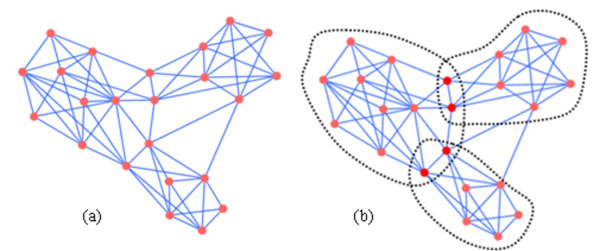
**The protein complex identified by algorithm CPM and the real protein complexes.** The left panel Fig.2 (a) depicts the protein complex identified by algorithm CPM with size 23 when using clique percolation parameter *k*=3. The real protein complexes what Fig.2 (a) correspond to are shown in the right panel of this figure Fig.2 (b).

In recent years, some researches have found that most important biological processes such as signal transduction, cell-fate regulation, transcription and translation involve more than four but much fewer than hundreds of proteins. Most relevant processes in biological networks correspond to the meso-scale (5-25 genes or proteins) [18,19]. Therefore, we expect that the large-scale of *k*-clique communities identified by algorithm CPM could be decomposed into multiple relatively dense subgraphs as the protein complexes with some special biological significance more effectively, more precisely and more comprehensively. In Fig.2, we use a schematic network to display protein complexes which detected by algorithm CPM and the ones in real protein-protein networks. Fig. 2(a) depicts the protein complex identified by algorithm CPM with size 23 when using clique percolation parameter *k*=3*.* In real protein-protein interaction networks, however, there should be three small overlapping protein complexes (Fig. 2(b)).

In algorithm CPM, each *k*-clique community is considered as a protein complex because of its dense internal links and sparse external linkage with other part of the protein-protein interaction networks. According to the relational biological characteristics and topological properties, the CPM method should be improved to identify protein complexes with more advantages such as meso-scale, high accuracy, more effectively and more comprehensively. In our approach, in order to achieve these advantages, we propose a new topological model by extending the definition of *k*-clique community of algorithm CPM and introduced distance restriction. Therefore, the scale of clusters identified by our approach is restricted by distance constraint and protein complexes considered as clusters satisfying distance limits.

Our new topological structure of identified clusters is based on the observation that a typical member in a cluster is linked to many other members, but not necessary to all other vertices in the cluster. In other words, our new topological structure of identified cluster can be interpreted as a union of small complete (fully connected) subgraphs that share vertices. We could definition the identified cluster as the union of all maximal cliques that satisfying the distance restriction and that can be reached from each other through a series of adjacent maximal clique (where two maximal cliques are said to be adjacent if they share *N* vertices). In this definition, the distance is represented by the diameter of the identified cluster (i.e., the largest length of a length of shortest path between a pair of vertices in the union of all maximal cliques).

In the following discussion, we donate by *U* and *V* basic cluster units, and by *C_c(U, V)_* the number of common vertices between basic cluster units *U* and *V,* by *C_l(U, V)_* the largest length of a length of shortest path between a pair of vertices in the union of *U* and *V.* Because of our new topological model based on this two condition *C_c(U, V)_* and *C_l(U, V)_,* our discussion will be mainly focused on them.

#### Condition 1

In the definition of our new topological model, the identified cluster could be seen as the union of all maximal cliques that can be reached from each other through a series of adjacent maximal clique (where two maximal cliques are said to be adjacent if they share *N* vertices). This condition can be depicted by the following formula:

*C*_*c*(*U, V*)_ ≥ *N*        (1)

where *N* represents the common vertices between basic cluster units *U* and *V.*

#### Condition 2

In our new topological model, the identified cluster also should be satisfying the distance restriction. As mentioned above, the distance is represented by the diameter of the identified cluster. According to the small-world property of the protein interaction networks [27,28], this condition can be defined by the following formula:

*C*_*l*(*U, V*)_ ≤ *d*        (2)

where *d* represents the diameter of the identified cluster.

It is known to all from previous subsection that two *k*-cliques will be merging if and only if they share *k*-1 vertices in algorithm CPM. According to this underlying idea, it is obviously that the larger value of the clique percolation parameter *k* chose, the more difficult *k*-cliques merge. The vast majority of predicted clusters are sole *k*-cliques. By the experimental analysis, the predicted clusters identified by this extending rule with large value of *k* are inefficient and incomplete. Reversely, the smaller value of *k* chose, the easier *k*-cliques merge and the scale of predicted clusters will be huger. That is why we obtain excessive huge clusters with 865 vertices and 4508 pair interactions in algorithm CPM when using the clique percolation parameter *k*=3*.* Generally speaking, the predicted clusters identified by this extending rule with small value of *k* are excessive large and low accuracy. In order to overcome this shortcoming of algorithm CPM, we define the novel extending rule as two maximal cliques will merge if they share *N* vertices. The number of common vertices *N* defined by the following formula:

*N* = *MIN*(|*U*|,|*V*|) – 1        (3)

where *|U|* and *|V|* are the size of basic cluster unit *U* and *V.*

In literature [18], Li *et al.* analyzing the topology of complex in the protein interaction network of *Saccharomyces cerevisiae.* Of the 216 gold standard protein complexes, 118 are connected (a protein complex is connected if there is a path connecting every pair of vertices in the complex). They have found that 94.91% of the connected complexes and 82.66% of the non-connected complexes have their diameter bounded 2. Furthermore, they found that 99.15% of the connected complexes and 93.88% of the non-connected complexes have their average shortest path length bound by 2. It is known to all this fact matches the observation that the protein interaction networks have the small-world property [27,28]. This analysis on the statistical data shows that the length of the shortest path between each pair of vertices in most of the complexes is bounded by 2. With this important conclusion, we believe that the diameter and the average shortest path length are important topological characteristic for detecting protein complexes. Therefore, in our novel topological model, we choose *d*=2 as the distance restriction condition, that is to say, we restrict arbitrary pair of vertices in identified clusters absolutely no more than 2.

**Figure 3:**
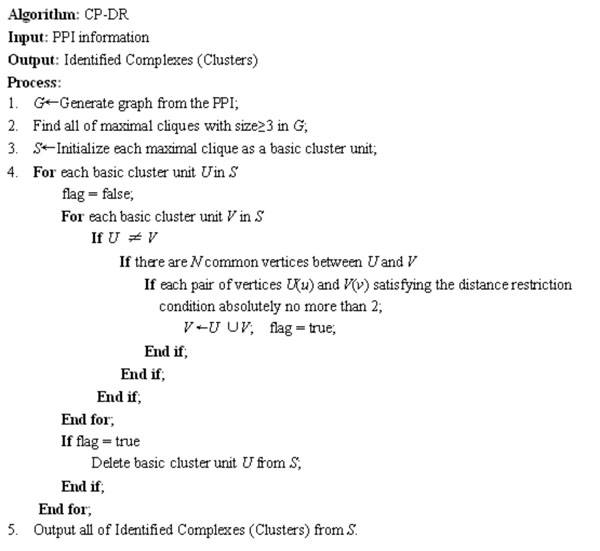
**The description of algorithm CP-DR.** The algorithm CP-DR (Clique Percolation Method based on Distance Restriction) based on a new topological model by extending the definition of *k*-clique community of algorithm CPM and introduced distance restriction.

According to the detailed depiction in characteristics of our new topological model, we propose a novel algorithm called CP-DR (Clique Percolation Method based on Distance Restriction) for identifying protein complexes based on clique percolation and distance restriction. The description of algorithm CP-DR is shown in Fig. 3.

As shown in Fig.3, algorithm CP-DR contains five major steps. The input to algorithm CP-DR is the protein-protein interaction information. According to the protein-protein interaction information, an undirected simple graph *G* (*V, E*) with proteins as vertices and protein interactions as edges is created firstly. And then, we search all of maximal cliques with size no less than 3 in *G.* Next, each maximal clique is initialized as a basic cluster unit. In the basic clusters unit collection, if there are *N* common vertices between any pairs basic cluster units (*U, V*) and the union of (*U, V*) satisfying the condition absolutely no more than 2 when basic cluster unit *U* is not same as basic cluster unit *V,* we will save the union of (*U, V*)*.* The first basic cluster unit will be deleted if and only if mergence appearance and all of comparison accomplishment. At last, all of identified clusters in *S* are exported.

In step 1 of algorithm CP-DR, the time complexity of protein-protein interaction information transformed into undirected simple graph is *O*(*m*)*.* Enumerating all maximal cliques with size no less than 3 is a NP-complete problem, and only non-polynomial time algorithms for solving it are known. It has an upper bound of *O*(*nmu*) in step 2. In step 3 of algorithm CP-DR, each maximal clique initialized as a basic cluster unit is *O*(*u*) for time complexity. In the core step of algorithm CP-DR, the time complexity has an upper bound of *O*(*u^2^s^3^*)*.* At last, the step of exporting all of identified clusters is *O*(*u*) for time complexity. This implies an upper bound of *O*(*nmu*+*u^2^s^3^*) for time complexity of algorithm CP-DR, where *n* is the number of nodes , *m* is the number of edges, *u* is the number of maximal cliques with no less than 3 in the graph and s is the size of largest maximal clique.

## Results and Discussions

To evaluate the suitability and validity of our proposed algorithm in identifying the overlapping protein complex in protein-protein interaction networks, we have used C++ language to implement algorithm CP-DR and download the overlapping protein complexes identification tool CFinder from http://angel.elte.hu/clustering/. The protein interaction network of *Saccharomyces cerevisiae* is downloaded from MIPS (Munich Information Center for Protein Sequences) database. We remove all the self-connecting interactions and repeated interactions. The final network includes 4546 yeast proteins and 12319 interactions. The average clustering coefficient of the final network is 0.4, the network diameter is 13, and the average vertex distance is 4.42. We applied the proposed algorithm CP-DR to this network.

In the following subsections, we will compare the predicted clusters with the known complexes, analyze the *sensitivity, specificity* and *f-measure* of the algorithm CP-DR, calculate the overlapping rate of the predicted clusters, and evaluate the significance of the predicted clusters. We will also compare the algorithm CP-DR to the clique percolation method CPM in their performance on these measures.

### Comparison with the known complexes

To evaluate the effectiveness of the algorithm CP-DR in detecting protein complexes, we compare the predicted overlapping structures produced by this algorithm with known protein complexes in MIPS yeast complex database. There are 216 manually annotated complexes considered as the gold standard data that each consists of two or more proteins. The largest complex contains 81 proteins, the smallest complex contains 2 proteins, and the average size of all the complexes is 6.31. Here, we use the same scoring scheme used in [17-20] to determine how effectively a predicted overlapping structure (*Pc*) matches a known complex (*Kc*)*.* The *overlapping score OS(Pc, Kc)* between a predicted overlapping structure *Pc* and a known complex *Kc* is calculated by the following formula:

        (4)

where *i* is the size of the interaction set of the predicted overlapping structure and the known complex, *|V_Pc_|* is the size of the predicted overlapping structure and |*V_Kc_*| is the size of the known complex.

A known complex *Kc* that has no proteins in a predicted overlapping structure *Pc* has *OS(Pc, Kc)* = 0 and a known complex *Kc* that perfectly matches a predicted overlapping structure *Pc* has *OS(Pc, Kc)* = 1. A known complex and a predicted overlapping structure are considered as a match if their overlapping score is equal to or larger than a specific threshold. Generally speaking, the more known complexes are matched by algorithm, the stronger identification ability algorithm has.

**Figure 4:**
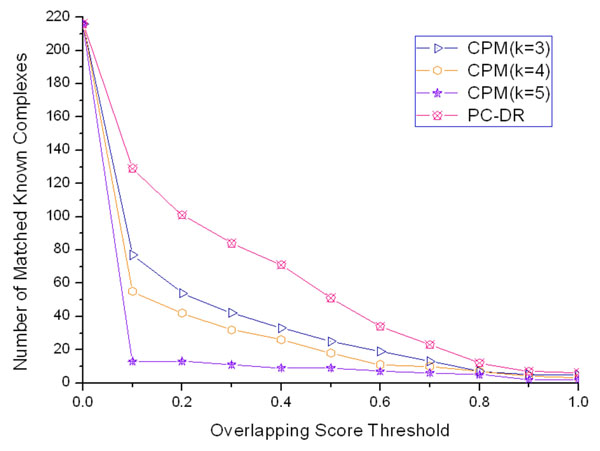
**Comparison of the predicted clusters with the known complexes.** The number of matched known complexes with respect to different overlapping scores threshold (from 0 to 1 with a 0.1 increment) for result data sets generated by algorithm CPM using different parameter values and algorithm CP-DR.

The numbers of matched known complexes with respect to different overlapping scores threshold (from 0 to 1 with a 0.1 increment) for result data sets generated by algorithm CPM using different parameter values and algorithm CP-DR are shown in Figure 4. In schematic diagram, the matched known complexes detected by algorithm CPM with the clique percolation parameter *k*=3, 4, 5 are shown by CPM (*k*=3), CPM (*k*=4) and CPM (*k*=5) respectively. It is CP-DR represents that the matched known complexes identified by our approach. From this schematic diagram, we understand that the numbers of matched known complexes with different overlapping scores threshold for identified protein complexes generated by algorithm CP-DR is significantly higher than that identified by algorithm CPM with arbitrary values of clique percolation parameter *k.* We can obtain the following conclusion by analysis Fig.4, the smaller clique percolation parameter *k* chose, the stronger identification ability in detecting known complexes algorithm CPM has. However, the identification ability of algorithm CPM is still further inefficient than that of algorithm CP-DR. We get the largest number of matched known complexes detected by algorithm CPM when clique percolation parameter *k*=3. However, the matched known complexes quantity which identified with different overlapping score thresholds decreasing with the value of *k* increasing. The reason of this phenomenon is that the basic topological unit *k*-cliques are decrease with the values of *k* increase. And the number of identified protein complexes is changed by the basic topological unit *k*-cliques quantity. The same conclusions had been provided by Zhang and Jonsson via taking advantage of CFinder analysis of protein-protein interaction network [21,22]. In our experiments, the identified protein complexes quantity detected by algorithm CPM are 178, 61, 18 corresponding to the values of clique percolation parameter *k*= 3, 4, 5 respectively. By the introduction of distance restriction in algorithm CP-DR, the numbers of identified protein complexes is 2013, which is far greater than the identified protein complexes quantity by algorithm CPM with any values of clique percolation parameter *k.* As shown in Figure 4, the numbers of matched known complexes with respect to different overlapping scores threshold for result protein complex sets generated by algorithm CP-DR is significantly higher than that identified by algorithm CPM.

In our experiment, we found that almost all of protein complexes identified by algorithm CPM could be accurately detected by algorithm CP-DR when protein complexes meeting the distance restriction condition. In addition, the introduction of distance restriction reasonable limits the size of protein complexes so that algorithm CP-DR could identify a large number of protein complexes with specific biological significance and biological functions more effectively, more precisely and more comprehensively. Table 1 shows some examples of protein complexes identified by algorithm CPM with the clique percolation parameter *k*=3 and algorithm CP-DR.

**Table 1:** Examples of protein complexes identified by algorithm CPM and algorithm CP-DR.

		CPM*(k*=3)		CP-DR	
		
Sequence	Known Complex	Size	*OS*(*Pc*, *Kc*)	Size	*OS*(*Pc*, *Kc*)
	YDR226c YER165w YKR002w				
Complex1	YMR061w YOL123w YGL044c			6	0.833
			
	YKR002w YMR061w YLR115w	13	0.089		
Complex2	YAL013c YLR277c YNL317w			9	0.854
	YJR093c YPR107c YDR301w				

	YPR041w YMR036c YBR079c				
Complex3	YNL244c	8	0.443	6	0.795
	YOR361w YMR146c YPL105c				
	YDR429c				

	YFL088c YKR068c YLR268w YIL004c				
Complex4	YML077w YDR407c YOR115c	18	0.150	13	0.923
	YMR218c				
	YBR254c YDR472w YGR166w				
	YDR246w				

In Table 1, the size of protein complexes identified by algorithm CP-DR is smaller than that identified by algorithm CPM, while the best matching extent of the protein complexes identified by algorithm CP-DR to known protein complexes are significant higher than that of algorithm CPM. The known protein complexes *Complex 1* and *Complex 2* are identified as an integral protein complex by algorithm CPM, but the real overlapping protein complexes could be detected by algorithm CP-DR and the matching extent are perfect.

**Figure 5:**
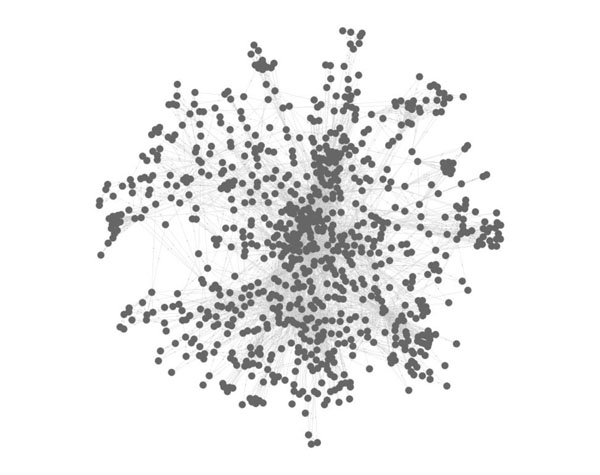
**The largest protein complex identified by algorithm CPM with clique percolation parameter *k*=3.** This huge complex contains 865 proteins, 4508 pairs of interactions, which involves approximate a quarter of protein vertices and one third interactions of the protein-protein interaction network.

**Figure 6:**
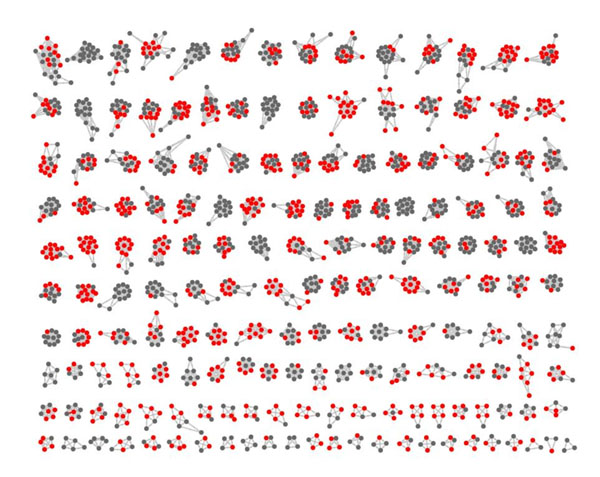
**A section of predicted complexes by algorithm CP-DR with *OS(Pc, Kc)* ≥0.2.** All of proteins and interactions are included in Fig.5. The red vertices represent overlapping proteins among these 172 protein complexes and the gray vertices represent non-overlapping proteins.

**Figure 7:**
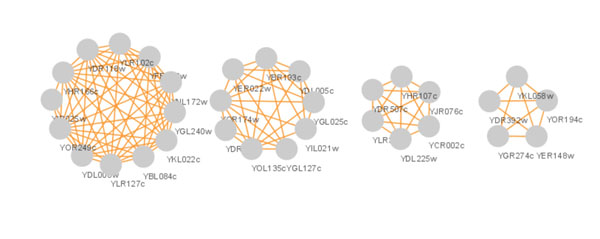
**Examples of protein complexes identified by algorithm CP-DR.** There are four protein complexes identified by algorithm CP-DR, which size respectively corresponding to 12, 9, 6, 5, best matching to known protein complexes *OS(Pc, Kc)* corresponding to 0.917, 0.559, 0.595, 1 respectively.

In our algorithm CP-DR, the size of protein complex is mainly restricted by distance constraint. It is precisely because of the introduction of distance restriction that the identified protein complexes with higher matching extent to known protein complexes and more prominent biological significance. A huge protein complex identified by algorithm CPM with clique percolation parameter *k*=3 is given in Fig.5. The complex, which involves approximate a quarter of protein vertices and one third interactions of the protein-protein interaction network, contains 865 proteins, 4508 pairs of interactions and the best matching extent *OS*(*Pc,Kc*) far less than 10^-3^. When we apply algorithm CP-DR to the same protein-protein networks, we indentify 1710 protein complexes which protein vertices and interactions included in the hugest protein complex identified by algorithm CPM. In Fig.6, there are 172 protein complexes which is a section of complexes with *OS*(*Pc,Kc*)≥0.2. In Fig.7, there are four protein complexes identified by algorithm CP-DR, which size respectively corresponding to 12, 9, 6, 5, best matching to known protein complexes *OS*(*Pc,Kc*) corresponding to 0.917, 0.559, 0.595, 1 respectively. In addition, we could obtain protein complexes 162, 348, 567, 689 respectively when the matching extent threshold *OS*(*Pc,Kc*) respectively no less than 0.5, 0.4, 0.3, 0.2 in our experiment. The result illustrate that the complexes, which identified by distance-based constraint restriction, comparing to what detected by algorithm CPM are more accurately, more efficiently and more comprehensively.

### Specificity and Sensitivity

*Sensitivity* and *specificity* are two important aspects to estimate the performance of algorithms in detecting protein complexes [20]. *Sensitivity* is the fraction of the true-positive predictions out of all the true predictions, defined by the following formula:

        (5)

where *TP* (true positive) is the number of the predicted complexes matched by the known complexes with *OS(Pc,Kc)* ≥0.2, and *FN* (false negative) is the number of the known complexes that are not matched by the predicted complexes. *Specificity* is the fraction of the true-positive predictions out of all the positive predictions, defined by the following formula:

        (6)

where *FP* (false positive) equals the total number of the predicted clusters minus *TP.* According to the assumption in [20], a predicted complex and a known complex are considered to be matched if *OS(Pc, Kc)* ≥0.2. Here, we also use 0.2 as the matched overlapping threshold.

Another integrated method, called the *f-measure,* has been used in [23], which is defined as follows:

        (7)

The *specificity, sensitivity* and comprehensive evaluation *f-measure* of algorithm CPM and algorithm CP-DR have been compared in Table 2.

**Table 2:** Comparison of algorithm CP-DR and algorithm CPM in Sensitivity, Specificity and f-measure.

Algorithm	Parameter	*Sn* ( Sensitivity )	*Sp* ( Specificity )	*F*-measure
CP-DR		0.872787611	0.391952310	0.540966747

	*k*=3	0.213592233	0.247191011	0.229166667
CPM	*k*=4	0.155339806	0.524590164	0.239700375
	*k*=5	0.092592593	0.722222222	0.164141415

As is known to all from Table 2, the sensitivity of algorithm CP-DR is greater than 0.87. This result shows that the number of detected complexes, *TP,* which matched by the known complexes with *OS(Pc,Kc)* ≥0.2 is significant greater than the unidentified complexes, *FN,* in the same threshold values. Therefore, there is a conclusion that the higher sensitivity correspond to lager *TP* and smaller *FN.* The conclusion also indicated that the detected complexes detected by algorithm CP-DR are greater reliability. The greatest sensitivity of algorithm CPM at arbitrary parameters is 0.213592233, only a quarter of sensitivity of algorithm CP-DR. As is well-known, Specificity is the fraction of the true-positive predictions out of all the positive predictions. The specificity of algorithm CP-DR is greater than 0.39, meanwhile, the specificity of algorithm CPM with arbitrary parameters *k* are greater than 0.2. Specifically, the algorithm CPM specificity is significant greater than that of algorithm CP-DR when the clique percolation parameter *k* ≥4. Though the specificity of algorithm CPM is higher than that of algorithm CP-DR, it is far less that the number of complexes identified by algorithm CPM compare to the known complexes quantity. In addition, because of the reference set MIPS is incomplete, some predicted complexes that may be true complexes could be regarded as false positives *(FP)* if they do not match with the known complexes. Nevertheless, it is still reasonable to consider a method more effective if it identifies more known complexes. From Table 2, we found the information that the comprehensive evaluation *f-measure* of algorithm CP-DR is more than twice the algorithm CPM. These results illustrate that the performance of algorithm CP-DR is more excellent in the protein complexes identification.

### Overlapping Rate Analysis

#### Definition 1

Overlapping Rate: In undirected graph *G*, the average occurrence times of each vertex v in all of induced subgraphs.

According to the definition, we calculate overlapping rate defined by the following formula:

        (8)

where *k_v_* is the number of occurrences of each vertex v in all of predicted complexes, *N_i_* is the size of predicted protein complexes, and *S* is all of identified protein complexes collection.

**Figure 8:**
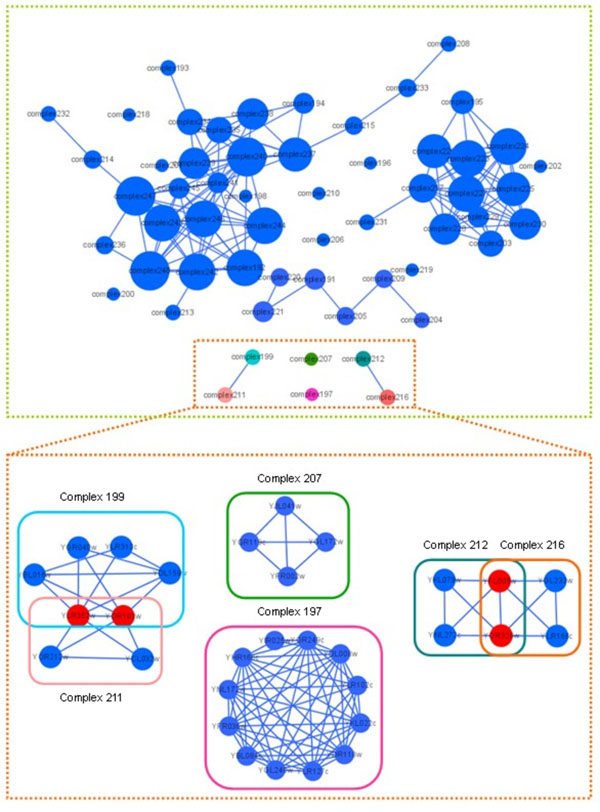
**Overlapping complexes identified by algorithm CP-DR.** According to the protein complexes existing overlap or not, we construct *Complex-Complex Interactions* with 58 members of 1710 protein complexes which include in the hugest protein complex identified by algorithm CPM.

Since each protein might be involved in multiple biological processes in the real protein-protein interaction networks, that is to say it might belong to several protein complexes, it is necessary to decompose protein-protein interaction networks into overlapping nested structures. Moreover, many researches have proved that this measure is consistent with the practical situation. In our paper, the protein complex identified by algorithm CPM containing 685 vertices and 4508 pair interactions corresponds to 1710 protein complexes detected by our approach. In order to analyze the overlapping rate, we selected 58 members of 1710 protein complexes. According to the protein complexes existing overlap or not, we could construct *Complex-Complex Interactions.* The experiment result has been shown in Fig.8 and the size of vertices in schematic diagram represents the degree of each complex. The vast majority of complexes satisfying distance restriction are overlapping. There is a phenomenon that one complex overlap with a number of complexes (Fig.8, upper panel). In addition, there are some isolated protein complexes. At the bottom panel of Fig.8, there are six complexes matched by the known complexes with *OS*(*Pc,Kc*) greater than 0.8. We found that complex199 and complex211 are overlapping as the same as complex211 and complex216. However, complex207 and complex197 are isolate respectively.

By the analysis protein complexes detected by algorithm CP-DR and algorithm CPM, we found that a vast majority of proteins only subordinate one or two complex. The situation of three or more protein complexes contain a same protein is rare.

Table 3 shows the overlapping rate of complexes identified by algorithm CPM is closely linked to clique percolation parameter *k.* When the clique percolation parameter *k*=3, the complexes overlapping rate is greatest, *OR*=1.192. Furthermore, the overlapping rate will decrease with the *k* increase. Finally, the overlapping rate will equal to one, that is to say, all of protein complexes are isolate respectively. The reason is that the basic topological unit *k*-cliques are decrease with the clique percolation parameter *k* increase. The number of predicted complex would reduce to one if the value of k increased to a certain extent. The overlapping rate is *OR* greater than 2.1 by analysis protein complexes predicted by algorithm CP-DR. Moreover, the rate of *OR*>1 of protein complexes predicted by algorithm CP-DR significant greater than that of algorithm CPM.

**Table 3:** Average overlapping rate of protein complexes identified by algorithm CP-DR and algorithm CPM.

	CP-DR	CPM(*k*=3)	CPM(*k*=4)	CPM( *k*=5)
Overlapping Rate	2.103	1.192	1.115	1.093

*OR*>	33.613%	13.843%	10.526%	9.685%

### Function Enrichment Analysis

In order to detect the functional characteristics of the predicted complexes, we compare the predicted complexes with known functional classification. The *P-value* based on hypergeometric distribution is often used to estimate whether a given set of proteins is accumulated by chance. It has been used as criteria to assign each predicted complex a main function [2,17]. Here, we also calculate *P-value* for each predicted complex and assign a function category to it when the minimum *P-value* occurs. The *P-value* is defied as follows [24,25]:

        (7)

where *N* is the total number of vertices in the network, *C* is the size of the predicted complex, *F* is the size of a functional group, and *k* is the number of proteins of the functional group in the predicted complex. As is well-known, the smaller difference between *P-value* and 0, the smaller possibility of protein complex possesses such function by chance and the larger possibility of protein complex encompasses special biological significance. Generally speaking, the main function of protein complexes corresponds to the minimum *P-value.* Therefore, we could predict the functions of unknown proteins by conferring the functions with the minimum *P-value* to identified complexes. The functional classification of proteins used in our paper was collected from the MIPS Functional Catalog (FunCat) database. FunCat [26] is an annotation scheme of tree-like structure for the functional description of proteins.

There are 1896 predicted protein complexes match with the known functional categories with *P-value* less than 0.01. Under the same conditions, there is 1823 predicted protein complexes match with *P-value* less than 0.001. Meanwhile, only 128 identified protein complexes by using the clique percolation parameter *k*=3 match with *P-value* less than 0.01, and 121 complexes match with *P-value* less than 0.001 using the clique percolation parameter *k*=3*.* By the above function enrichment analysis, we could understand, the capacity of algorithm CP-DR identifying protein complexes with special biological significance is much higher than that of algorithm CPM.

According to the *P-value* definition, the *P-value* is relational closely to the protein complex scale. Generally speaking, the larger size of protein complexes corresponds to relativity the smaller *P-value.* The size of protein complexes predicted by algorithm CP-DR is restricted by distance constraint, while the *P-value* of predicted protein complexes has not apparent change. For instance, the *-log(P-value)* value of a protein complex predicted by algorithm CPM containing 18 proteins is 26. However, this complex is decomposed into three protein complexes by algorithm CP-DR and the *-log*(*P-value*) value of each protein complexes corresponding to 23, 17, 14 respectively. There is full functional association information of two predicted complexes in Table 4. The one is introduced from Table 1, named Table 1(13), the other is introduced from Figure 8, called Fig.8(complex197). The maximum -log(*P-value*) of protein complex Table 1(13) detected by algorithm CP-DR with size 13 is 23. All these proteins in the complex correspond to the functional categories of 20.09.07.03 (*ER to Golgi Transport*) completely consistency. The maximum -*log*(*P-value*) of protein complex Fig.8(complex197) identified by algorithm CP-DR with size 12 is also 23. All the proteins in this predicted cluster have at least four functional categories completely consistency which are 10.03.01.01.11 (*Mitosis M Phase*), 14.07 (*Protein Modification*), 14.13.01.01 *(Proteasomal Degradation (Ubiquitin/Proteasomal Pathway)*), 16.01 (*Protein Binding*) and so on. All proteins except YGL240w correspond to 14.10 (*Assembly of Protein Complexes*) and 16.19.03 (*ATP Binding*) high consistency. As the fact that proteins in the same complex are of similar functions, we could predict new functions for known proteins. Thus, we can predict that the function known protein YGL240w is also involved in the functional categories 14.10 (*Assembly of Protein Complexes*) and 16.19.03 (*ATP Binding*) in real biological process, too. In fact, researchers have found the protein YGL240w absolutely has these functions. Furthermore, the functions annotated only for some individual proteins in the complex could also provide clues for studying protein function.

**Table 4:** Functional annotation of predicted complexes in Table 1(13) and Figure 8 (the code represents functional category)

Complexes	ORF				Protein functional categories				
Table 1 (13)	YLR268w				20.09.07.03	20.09.07.05	20.09.07.27		
	YKR068c				20.09.07.03				
	YML077w				20.09.07.03				
	YFL038c			14.10	20.09.07.03				
	YGR166w	01.05.25			20.09.07.03				
	YDR108w		10.03.02		20.09.07.03			43.01.03.09	
	YBR254c				20.09.07.03				
	YDR246w				20.09.07.03				
	YDR407c				20.09.07.03				
	YDR472w				20.09.07.03				
	YMR218c				20.09.07.03				
	YOR115c				20.09.07.03				
	YIL004c				20.09.07.03		20.09.07.27		

Figure 8 (complex197)	YBL084c		10.03.01.01.11	14.07.05	14.10	14.13.01.01	16.01	16.19.03	
	YDL008w		10.03.01.01.11	14.07.05	14.10	14.13.01.01	16.01	16.19.03	
	YDR118w		10.03.01.01.11	14.07.05	14.10	14.13.01.01	16.01	16.19.03	
	YFR036w		10.03.01.01.11	14.07.05	14.10	14.13.01.01	16.01	16.19.03	
	YGL240w		10.03.01.01.11	14.07		14.13.01.01	16.01		
	YHR166c		10.03.01.01.11	14.07.05	14.10	14.13.01.01	16.01	16.19.03	42.04
	YKL022c		10.03.01.01.11	14.07.05	14.10	14.13.01.01	16.01	16.19.03	
	YLR127c		10.03.01.01.11	14.07.05	14.10	14.13.01.01	16.01	16.19.03	
	YNL172w		10.03.01.01.11	14.07.05	14.10	14.13.01.01	16.01	16.19.03	
	YOR249c	10.01.09.05	10.03.01.01.11	14.07.05	14.10	14.13.01.01	16.01	16.19.03	
	YLR102c		10.03.01.01.11	14.07.05	14.10	14.13.01.01	16.01	16.19.03	
	YIR025w		10.03.01.01.11	14.07.05	14.10	14.13.01.01	16.01	16.19.03	

The Code of Figures Represent Function: 01.05.25: regulation of C-compound and carbohydrate metabolism; 10.01.09.05: DNA conformation modification; 10.03.01.01.11: mitosis M phase; 10.03.02: meiosis; 14.07.05: modification by ubiquitination, deubiquitination; 14.10: assembly of protein complexes; 14.13.01.01: proteasomal degradation (ubiquitin/proteasomal pathway); 16.01: protein binding; 16.19.03: ATP binding; 20.09.07.03: ER to Golgi transport; 20.09.07.05: intra Golgi transport; 20.09.07.27: vesicle fusion; 42.04: cytoskeleton/structural proteins; 43.01.03.09: development of asco-basidio- or zygospore.

## Conclusions

It is believed that identification of protein complexes is useful to explain certain biological progress and to predict functions of proteins. In this paper, we extended the definition of *k*-clique community of algorithm CPM, introduced distance restriction, proposed a new topological model for protein complexes and developed an algorithm CP-DR to identify protein complexes in large protein interaction networks based on the proposed topological model. Interaction networks are represented by undirected simple graphs and we generate predicted clusters in the networks by using clique percolation and distance restriction. The algorithm CP-DR could generate overlapping protein complexes, which is consistent with the fact that many of the known protein complexes are overlapping. Interesting questions for further research include how many functions a protein can have, how many processes a protein can participate in, and how heavily two protein complexes should overlap with each other.

We applied the algorithm CP-DR to the protein interaction network of *Sacchromyces cerevisiae.* Many well-known complexes were detected in the protein interaction network. We tested the *sensitivity, specificity* and *f-measure* of our algorithm. The results have shown that our algorithm is suitability and efficiency in the protein interaction networks. We also predict the functions for un-characterized proteins and predicted new functions for the known proteins by minimizing the *P-values* of the predicted clusters. Our algorithm can thus be used to identify new protein complexes in protein interaction networks of various species and to provide references for biologists in their research on protein complexes.

## Methods

The protein interaction network of *Saccharomyces cerevisiae* is downloaded from MIPS (Munich Information Center for Protein Sequences) database. We remove all the self-connecting interactions and repeated interactions. The final network includes 4546 yeast proteins and 12319 interactions. We also collect from the MIPS database protein complexes annotated for *Sacchromyces cerevisiae.* We discarded those consisting of only one protein and the final remaining 216 manually annotated complexes are considered as the gold standard data. The largest complex contains 81 proteins, the smallest complex contains 2 proteins, and the average size of all the complexes is 6.31. We download the overlapping protein complexes identification tool CFinder from http://angel.elte.hu/clustering/. The proposed algorithm CP-DR has been implemented in C++.

## Competing interests

The authors declare that they have no competing interests.
